# Clinical outcomes and functional analysis of third space robotic and endoscopic cooperative surgery versus laparoscopic wedge resection for gastric submucosal tumours: a propensity score-matched study

**DOI:** 10.1007/s13304-021-01014-6

**Published:** 2021-03-11

**Authors:** Feiyu Shi, Gaixia Liu, Qi Sun, Haowei zhang, Hongtao Wu, Xiaobin Xue, Yingchao Li, Junjun She

**Affiliations:** 1 Department of General Surgery, The First Affiliated Hospital of Xi’an Jiao Tong University, No. 277, Yanta West Road, Xi’an, 710061 Shaanxi China; 2 Department of Talent Highland, The First Affiliated Hospital of Xi’an Jiao Tong University, No. 277, Yanta West Road, Xi’an, 710061 Shaanxi China; 3 Center for Gut Microbiome Research, Med-X Institute, The First Affiliated Hospital of Xi’an Jiao Tong University, Xi’an, Shaanxi China; 4 Department of Gastroenterology, The First Affiliated Hospital of Xi’an Jiao Tong University, No. 277, Yanta West Road, Xi’an, 710061 Shaanxi China

**Keywords:** Gastrointestinal submucosal tumours, Third space robotic and endoscopic cooperative surgery, Laparoscopic wedge resection, Clinical outcomes, Gastrointestinal function

## Abstract

Third space robotic and endoscopic cooperative surgery (TS-RECS) is a novel minimally invasive surgery for resecting gastric submucosal tumours (GSMTs), which could accomplish the completely oncological curability and maximal functional preservation. This study investigated the clinical outcomes and gastrointestinal function after TS-RECS versus laparoscopic wedge resection (LWR) for GSMTs. This was a single-centre retrospective study that included 130 patients with GSMTs who underwent LWR or TS-RECS from 2013 to 2019. To overcome selection biases, we performed propensity score matching (1:1) using seven covariates that could impact the group assignment and outcomes. Then, the clinical outcomes and gastrointestinal function in the LWR and TS-RECS groups were compared in a matched cohort. Among the 130 enrolled patients, 96 patients underwent LWR, and 34 underwent TS-RECS and were matched into 30 patients for each group. There was no significant difference in the operation time between the two groups (*P* = 0.543). However, the TS-RECS group had significantly less blood loss (20,5–100 vs 95,10–310 ml, *P* < 0.0001) and better postoperative recovery in terms of time to oral intake (2,2–4 vs 3,2–6 days, *P* < 0.0001) and postoperative hospital stay (5,4–10 vs 8.5,5–16 days, *P* < 0.0001) than the LWR group. The severity and frequency scores of postoperative gastrointestinal symptoms in the TS-RECS group were significantly lower than those in the LWR group. The median follow-up period was 24 months (10–60 months) in the LWR group and 18 months (10–27 months) in the TS-RECS group, and there was in total a single recurrence in the LWR group. TS-RECS appears to be a technically safe and effective surgery with preservation of gastrointestinal function for resection of GSMT resection.

## Introduction

Due to the rare possibility of lymph node metastasis in gastric submucosal tumours (GSMTs), including gastrointestinal stromal tumours (GISTs), local R0 resection without lymph node dissection is always considered the standard treatment for patients with GSMTs^1^,^2^. During the past decades, the surgical management of GSMTs has evolved towards minimal invasiveness. Since the first study of laparoscopic surgery for GSMTs was reported in 1992^3^, laparoscopic resection has been gradually applied to treat GSMTs worldwide because of its lower invasiveness, lower postoperative morbidity, shorter hospital stay and comparable oncology prognosis compared with the open approach^4^. Laparoscopic wedge resection (LWR) is one of the most common laparoscopic approaches for GSMT resection and has been confirmed by many studies regarding its feasibility, safety and effectiveness^5^–^7^.

However, there are several limitations in the LWR technique. First, when LWR is used for removing the GSMTs, especially for the type of intragastric growth pattern, a certain amount of innocent gastric wall that enwraps the tumour would be resected with the lesion together^8^. These excessive resections may cause severe gastric malformation, which would impair postoperative gastrointestinal function. Second, the full-thickness incisions caused by LWR may increase the risk of postoperative intra-abdominal infections or gastrointestinal tract leakage. Third, for some tumours located at challenging anatomical sites, such as the oesophagogastric junction, pyloric region or lesser curvature, the LWR procedure is technically demanding and may increase the risk of some postoperative complications, including inlet or outlet stenosis or delayed gastric emptying^8^,^9^.

To address the above problems, we developed a novel surgical procedure, termed third space robotic and endoscopic cooperative surgery (TS-RECS), to resect GSMTs and reported its clinical feasibility and safety in a prospective study^10^. We believe that this procedure, which combines the advantages of both endoscopic techniques and robotic surgery, can dissect the tumour entirely with minimal negative margins and simultaneously preserve the intact mucosal layer of the stomach. In addition, TS-RECS could be performed independently on the location of tumours due to technical advantages. With this technique, we could minimize surgical invasiveness and maximally preserve the anatomical integrity and function of the stomach. In our opinion, these benefits would greatly reduce the negative impact of surgery on gastrointestinal function. However, limited information is available to prove the superiority of TS-RECS in terms of postoperative gastrointestinal function.

Therefore, this study aimed to investigate the clinical and functional outcomes of TS-RECS compared with LWR for GSMT resection in a propensity score-matched cohort.

## Methods

### Patients and study design

**Fig. 1:**
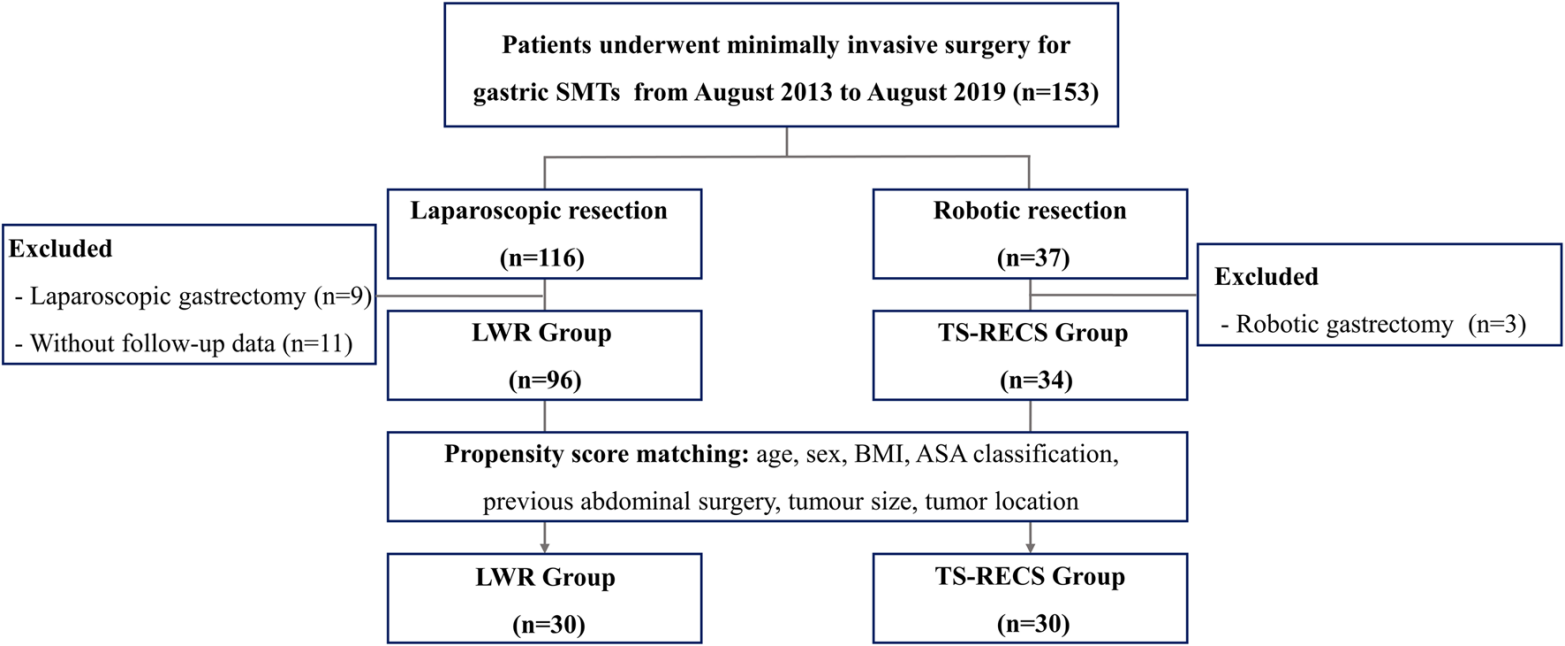
Flowchart of patient screening and grouping. SMTs, submucosal tumours; LWR, laparoscopic wedge resection; TS-RECS, third space robotic and endoscopic cooperative surgery

This was a single-centre retrospective study based on a prospectively collected database of upper GI tumours in our institution. A total of 153 patients who underwent minimally invasive surgery (laparoscopic surgery and robotic surgery) for GSMTs at The First Affiliated Hospital of Xi'an Jiao Tong University between June 2013 and June 2019 were identified. The exclusion criteria were patients who underwent gastrectomy (total/subtotal gastrectomy) for any reason or purpose and patients without follow-up data. According to the above criteria, 130 patients were enrolled in this study and divided into two groups according to the surgical method (LWR group vs TS-RECS group). The flowchart of patient screening and grouping is depicted in Fig. 1. We used propensity score matching (PSM) analysis in these patients to reduce selection bias, which may impact the comparison of clinical outcomes and functional analyses^11^. This study was approved by the Institutional Review Board of The First Affiliated Hospital of Xi'an Jiao Tong University (XJTU1AF2018LSK-168).

### Surgical procedures

All surgical procedures (laparoscopic wedge resection and robotic resection) were performed by three skilled surgeons with similarly high levels of minimally invasive surgical experience. All endoscopic procedures were performed by a single experienced endoscopist. The procedure details of LWR and TS-RECS were performed according to previously reported methods^5^–^7^,^10^. The TS-RECS technique was performed in accordance with the following six steps: (i) setting up the da Vinci surgical system; (ii) confirmation of the tumour location; (iii) local blood vessel preparation and division of adhesions (if necessary); (iv) establishment of the third space by endoscopic submucosal injection; (v) robotic submucosal dissection of the tumour; and (vi) closure of the seromuscular incision.

### Data collection and function assessment

We collected the following clinical data from our prospective database: 1. demographic characteristics including age, sex, BMI; 2. clinicopathologic characteristics including American Society of Anesthesiologists (ASA) classification, clinical symptoms, previous abdominal surgery, tumour size, tumour location, pathological diagnosis, mitotic index and Fletcher risk classification (for GISTs); 3. clinical outcomes including operative time, blood loss, conversion, en bloc resection, resection margin, time to oral intake, postoperative hospital stay, postoperative complications (Clavien–Dindo Grade ≥ 2), C-kit mutational status (CD117) and Ki-67 labelling index; and 4. follow-up data including postoperative endoscopy record, tumour recurrence, metastasis and death.

In addition, follow-up assessment of gastrointestinal function was performed prospectively with a well-validated and self-administered questionnaire, namely, the Gastrointestinal Symptom Rating Scale (GSRS)^12^,^13^. According to the study reported by E. Dimends et al.^13^, 15 symptoms of GSRS were grouped into 5 dimensions (reflux syndrome, abdominal pain syndrome, indigestion syndrome, constipation syndrome and diarrhoea syndrome) for further analysis. Based on the severity, frequency and impact on daily life of symptoms, all items had a score scale from 0 to 3 points. Zero points indicated no symptoms or normal conditions, and 3 points indicated the most pronounced symptoms.

### Follow-up

Patients who underwent LWR and TS-RECS were generally followed up at 3 months and 6 months after surgery (at least one endoscopy within the first six months) and asked to complete the GSRS questionnaire through telephone or outpatient service. Then, for GIST patients with very low risk or low risk, endoscopy and CT scans were performed every 6 months for at least five years. GIST patients with intermediate or high risk were followed up every 3 to 6 months for the first 3 years and every 6 months thereafter.

### Statistical analysis

Continuous variables are shown as the mean ± standard deviation (normally distributed) or median plus range (nonnormally distributed), and categorical variables are shown as the number of cases. Student’s t test or the Mann–Whitney U test was used to compare the differences in continuous variables, and the Chi-square test or Fisher’s exact test was used to compare categorical variables. To reduce selection bias and confounding variables, propensity score matching analysis was used to compare the LWR group and TS-RECS group. First, we selected seven covariates, including age, sex, BMI, ASA classification, previous abdominal surgery, tumour size and tumour location. Then, we estimated the propensity score by a logistic regression model based on these covariates. Finally, the patients in the TS-RECS group were matched at a 1:1 ratio with the patients in the LWR group using the nearest neighbour method with a maximum allowable calliper width of 0.2. Statistical analysis was performed using SPSS version 23.0 (IBM, Armonk, NY, USA), and PSM was performed using R Project. A two-tailed *P* < 0.05 was considered statistically significant.

## Results

### Clinicopathological characteristics of the patients

The clinicopathological characteristics of all enrolled patients are shown in Table 1. Before PSM, the mean age of patients in the LWR group was significantly older than that in the TS-RECS group (63.06 ± 9.24 years vs 56.88 ± 11.44 years, *P* = 0.002). The LWR group had a larger tumour size than the TS-RECS group (40, 14–90 mm vs 35,15–65 mm, *P* = 0.039). Moreover, there was also a significant difference in tumour location between the two groups (*P* = 0.016). After PSM, there was no significant difference in any clinicopathological characteristics between the LWR group and TS-RECS group (all *P* > 0.05).

**Table 1:** Baseline clinicopathologic characteristics of LWR group and TS-RECS group before and after PSM

Categories	Before PSM			After PSM		
	LWR group (n=96)	TS-RECS (n=34)	*P* value	LWR group (n=30)	TS-RECS (n=30)	*P* value
Age, years	63.06±9.24	56.88±11.44	0.002	57.43±10.53	57.73±11.47	0.916
Gender, n						
Male	54	20	0.842	20	19	0.787
Female	42	14		10	11	
BMI, kg/m^2^	22.96±2.70	22.04±1.87	0.069	22.79±2.57	22.05±1.93	0.216
ASA score, n						
I	57	20	0.918	18	17	0.834
II	35	12		11	11	
III	4	2		1	2	
Clinical symptoms, n						
No	76	24	0.308	26	25	0.718
Yes	20	10		4	5	
Previous abdominal surgery, n						
No	87	31	0.924	28	26	0.389
Yes	9	3		2	4	
Tumour size, mm	40 (14 - 90)	35 (15 - 65)	0.039	34 (15 - 90)	35 (15 - 60)	0.716
Tumour location, n						
Cardia	0	2	0.016	0	0	0.212
Upper-third	56	16		17	16	
Middle-third	28	11		6	11	
Low-third	12	3		7	3	
Pyloric	0	2		0	0	
Pathological diagnosis, n						
GIST	83	30	0.792	25	26	0.718
No GIST^a^	13	4		5	4	
Mitotic index^b^ (per 50 HPF), n						
≤5	72	27	0.573	23	24	0.513
6-10	8	3		1	2	
>10	3	0		1	0	
Fletcher risk classification^b^, n						
Very low risk	8	1	0.206	4	1	0.203
Low risk	50	24		16	21	
Intermediate risk	21	5		3	4	
High risk	4	0		2	0	
C-kit mutational status (CD117)^b^, n						
Positive	78	28	0.900	24	25	1.000
Negative	5	2		1	1	
Ki-67 labelling index^b^, n						
<10%	66	28	0.083	21	24	0.627
≥10%	17	2		4	2	

*BMI*, body mass index; *ASA*, American Society of Anesthesiologists; *GIST*, gastrointestinal stromal tumors; *LWR*, laparoscopic wedge resection; *TS-RECS*, the third space robotic and endoscopic cooperative surgery; PSM, propensity score matching

^a^No GIST included 2 lipomas, 5 schwannomas, 6 leiomyomas in LWR group, and 2 schwannomas and 2 leiomyomas in TS-RECS group

^b^Only for GIST

### Comparison of clinical outcomes between the LWR group and TS-RECS groups after PSM

The operation-related data and postoperative outcomes after PSM are presented in ***Table ******2*****.** The median operative time in the TS-RECS group was 115 (75–235 min), which did not increase significantly compared with 112.5 (70–280 min) for the LWR group (*P* = 0.543). Meanwhile, the TS-RECS group was associated with significantly less blood loss than the LWR group (20, 5–100 ml vs 95, 10–310 ml, *P* < 0.0001). In the TS-RECS group, the integrity of the mucosal layer was maintained in 96.7% (29/30) of the patients. No patient underwent conversion surgery, and en bloc resection with a negative surgical margin was performed in both groups. In terms of postoperative outcomes, the overall incidence of postoperative complications (Clavien–Dindo grade ≥ 2) was not significantly different between the TS-RECS group and the LWR group (*P* > 0.05). However, the TS-RECS group had a better postoperative recovery than the LWR group in terms of time to first oral intake (2, 2–4 days vs 3, 2–6 days, *P* < 0.0001) and postoperative hospital stay (5, 4–10 days vs 8.5, 5–16 days, *P* < 0.0001). In addition, we reviewed the endoscopic records of 54 patients (TS-RECS: 29; LWR: 25) within the first six months after surgery. There was no evidence of food residue in the TS-RECS group, whereas 2 positive cases were noted in the LWR group. One was a 76-year-old woman with a 40-mm-diameter GIST in the lower third of the stomach, and the other was a 63-year-old man with a 60-mm-diameter GIST in the middle third of the stomach.

**Table 2:** Comparison of clinical outcomes between LWR group and TS-RECS group after propensity score matching

Categories	LWR group (*n* = 30)	TS-RECS (*n* = 30)	*P* value
Operative time, min	112.5 (70–280)	115.0 (75–235)	0.543
Intraoperative blood loss, ml	95.0 (10–310)	20.0 (5–100)	0.000
Conversion, n	0	0	1.000
En bloc resection, n	30	30	1.000
Resection margin, n			
R0	30	30	1.000
R1	0	0	
Time to oral intake, days	3 (2–6)	2 (2–4)	0.000
Postoperative hospital stays, days	8.5 (5–16)	5.0 (4–10)	0.000
Complications, n	5	2	0.228
Pneumonia	0	1	
Abdominal incision infection	0	1	
Anastomotic bleeding	1	0	
Gastric emptying disorder	2	0	
Leakage	2	0	
Follow-up time, months	24 (10–60)	18 (10–27)	0.004
Recurrence, n	1	0	0.313
Recurrence-related death, n	0	0	1.000

*LWR* laparoscopic wedge resection, *TS-RECS* third space robotic and endoscopic cooperative surgery

With a median follow-up period of 24 months (10–60 months) in the LWR group and 18 months (10–27 months) in the TS-RECS group, there was in total a single recurrence in the LWR group (a 50-year-old man with high-risk GIST). No recurrence-related deaths have been reported to date.

### Comparison of gastrointestinal symptoms at 3 and 6 months after surgery between the LWR group and TS-RECS group

**Fig. 2:**
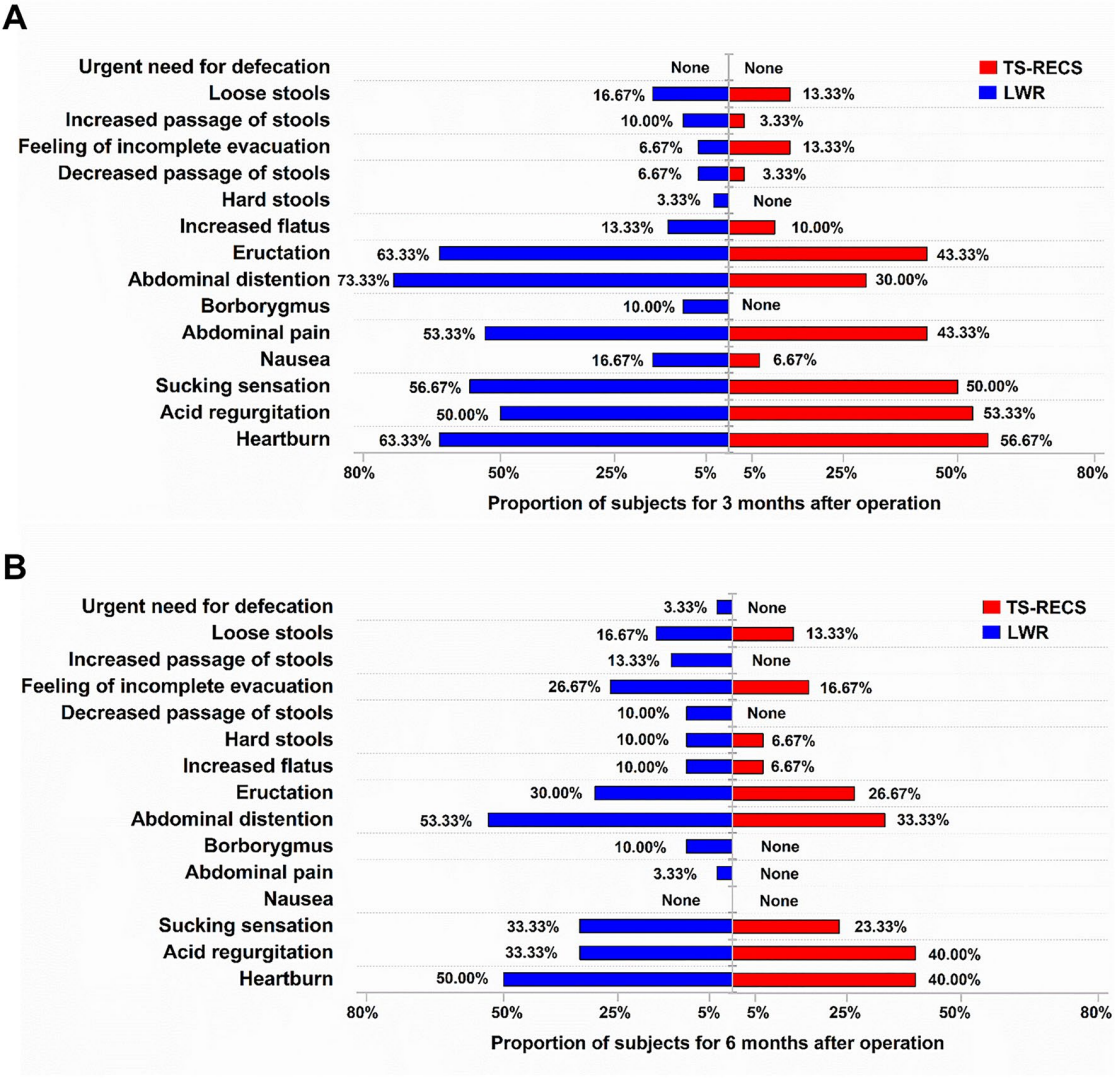
The frequency of 15 gastrointestinal symptoms as evaluated with the Gastrointestinal Symptom Rating Scale (GSRS) in the LWR and TS-RECS groups. **a** Frequency of 15 gastrointestinal symptoms in the LWR group (blue) and TS-RECS group (red) three months postoperatively. **b** Frequency of 15 gastrointestinal symptoms in the LWR group (blue) and TS-RECS group (red) 6 months postoperatively

The frequency of 15 gastrointestinal symptoms recorded in the GSRS is presented in Fig. 2a, b. Three months postoperatively, a total of 6 gastrointestinal symptoms afflicted more than half of the patients in the LWR group (heartburn: 63.33%; acid regurgitation: 50.00%; sucking sensation: 56.67%; abdominal pain: 53.33%; abdominal distention: 73.33%; eructation: 63.33%). For the TS-RECS group, there were only 3 gastrointestinal symptoms afflicting more than half of the patients (heartburn: 56.67%; acid regurgitation: 53.33%; sucking sensation: 50.00%) (Fig. 2a). At 6 months after surgery, there was still a higher frequency of gastrointestinal symptoms in patients in the LWR group, especially heartburn (50.00%) and abdominal distention (53.33%) (Fig. 2b).

**Fig. 3:**
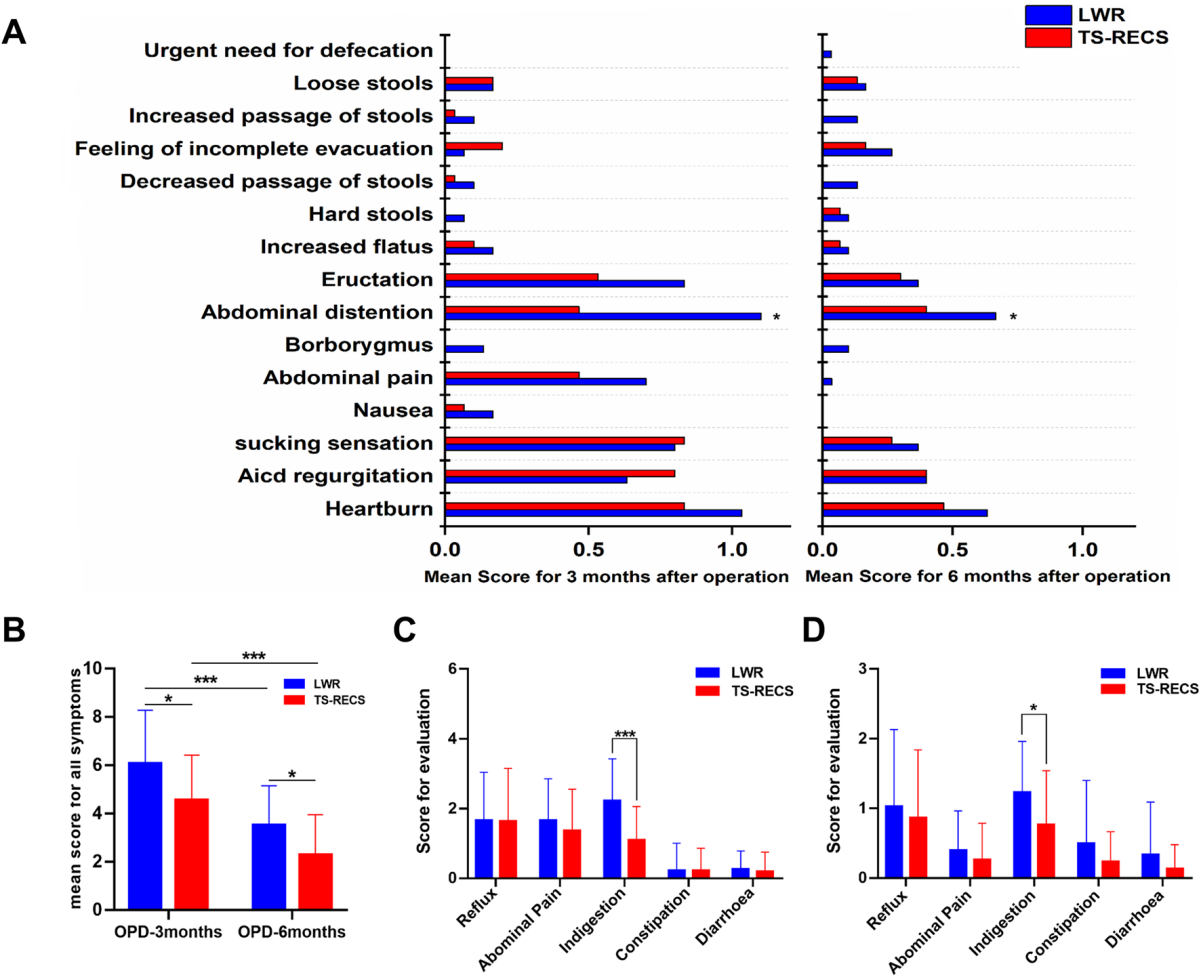
The score of 15 gastrointestinal symptoms as evaluated with the Gastrointestinal Symptom Rating Scale (GSRS) in the LWR and TS-RECS groups. **a** The mean score of 15 gastrointestinal symptoms in the LWR group and TS-RECS group 3 months and 6 months postoperatively. **b** Total mean GSRS scores of the LWR group and TS-TECS group 3 months and 6 months postoperatively. **c** GSRS scores of five symptom clusters in the LWR and TS-RECS groups 3 months postoperatively. **d** Five symptom cluster GSRS scores of the LWR and TS-RECS groups 6 months postoperatively. POD, postoperative day; ***, *P* < 0.0001; **, *P* < 0.001; *, *P* < 0.05

The mean score of 15 gastrointestinal symptoms is shown in Fig. 3a, which revealed that the patients in the LWR group suffered from more severe gastrointestinal symptoms at 3 months postoperatively, especially eructation, abdominal distention, abdominal pain and heartburn. Statistically significant differences between the groups were observed in abdominal distention (*P* < 0.05) (Fig. 3a). At 6 months after surgery, all symptoms in both groups were relieved, but the patients in the LWR group still had more serious abdominal distention and heartburn than those in the TS-RECS group.

The GSRS total scores and subgroup scores of the 5 dimensions are given in Table 3 and Fig. 3b to d. At 3 months after surgery, a statistically significant difference was found in the GSRS total scores between the two groups, with worse symptoms reported by patients in the LWR group than by the TS-RECS group (mean 5.97, 95% CI 5.14–6.79 vs mean 4.33, 95% CI 3.67–5.00, P = 0.003). Among the five dimensions, a statistically significant difference was found in indigestion syndrome between the two groups (LWR: mean 2.23, 95% CI 1.79–2.68 vs TS-RECS: mean 1.10, 95% CI 0.74–1.46, P < 0.0001), and no difference was found in other syndromes. At 6 months after surgery, a significant difference still existed between the LWR group and TS-RECS group (Table 3 and Fig. 3).

**Table 3:** The score of Gastrointestinal Symptom Rating Scale at 3 and 6 months after surgery in LWR group and TS-RECS group

		LWR			TS-RECS				*P* value
		Mean	95% CI		Mean	95% CI			
GSRS total	POD 3 months	5.97	5.14	6.79	4.33	3.67	5.00		0.003
	POD 6 months	3.50	2.86	4.14	2.33	1.68	2.99		0.012
Abdominal pain syndrome	POD 3 months	1.67	1.22	2.11	1.37	0.92	1.81		0.331
	POD 6 months	0.40	1.19	0.61	0.27	0.07	0.46		0.345
Indigestion syndrome	POD 3 months	2.23	1.79	2.68	1.10	0.74	1.46		0.000
	POD 6 months	1.23	0.96	1.51	0.77	0.48	1.06		0.019
Reflux syndrome	POD 3 months	1.67	1.15	2.18	1.63	1.07	2.20		0.929
	POD 6 months	1.03	0.62	1.44	0.87	0.5	1.23		0.536
Constipation syndrome	POD 3 months	0.23	-0.06	0.52	0.23	0.00	0.47		1.000
	POD 6 months	0.50	0.16	0.84	0.23	0.07	0.39		0.151
Diarrhoea syndrome	POD 3 months	0.27	0.07	0.46	0.20	-0.01	0.41		0.632
	POD 6 months	0.33	0.05	0.62	0.13	0.00	0.26		0.196

*POD* postoperative day, *GSRS* Gastrointestinal Symptom Rating Scale, *LWR* laparoscopic wedge resection, *TS-RECS* third space robotic and endoscopic cooperative surgery

## Discussion

Up to half of GSMTs have malignant potential, most commonly being GISTs^14^,^15^. Considering that GSMTs have a pseudocapsule and exhibit rapid growth but rarely present lymph node metastasis, wide resection margins and lymphadenectomy are unnecessary and not recommended. Therefore, an increasing number of researchers have suggested that GSMT resection be performed with minimal sacrifice of the innocent gastric wall to preserve the volume of the remnant stomach as much as possible, which could be beneficial for postoperative gastrointestinal function^8^,^9^. In accordance with this concept, we developed the novel technique TS-RECS^10^.

In the present study, we compared the clinical outcomes and postoperative gastrointestinal function of TS-RECS versus classical minimally invasive surgery (LWR) in a matched cohort. Considering our initial experience in TS-RECS, the indication criteria for this technique were limited to GSMTs originating from the muscularis propria (MP) with an intact capsule and a maximal transverse diameter ≤ 6.5 cm, independent of tumour location, which was slightly different from LWR. These differences could lead to some selection bias. Moreover, some differences in patient characteristics (e.g., age) could affect the comparisons of clinical outcomes and postoperative gastrointestinal function. Therefore, we used PSM with seven potential covariates (age, sex, BMI, ASA classification, previous abdominal surgery, tumour size and tumour location) that could impact the group assignment and outcomes, which allowed for more reliable statistical results. In addition, the experience level of surgeons could have a major impact on clinical outcomes and postoperative gastrointestinal functions. In our study, all TS-RECS procedures were performed by a single surgeon, and the LWR procedure was performed by three experienced surgeons with extensive experience in minimally invasive surgeries, including above-mentioned surgeon, which helped to decrease bias caused by surgical experience. Finally, in our matched cohort, our results revealed that TE-RECS was as safe and effective as LWR for GSMT resection and had some additional advantages in terms of procedural technique and preservation of organ function.

From a technical standpoint, robotic surgery does have some technical superiorities over laparoscopic surgery, such as flexible and precise multi-joint forceps, a tremor-filtering function and a high-quality 3-D^16^. Many studies have demonstrated that these technical merits could be translated into clinical advantages in stomach surgery, including less blood loss^17^, better postoperative recovery^18^ and reduced morbidity^19^. In our study, these technical superiorities were illustrated by the more favourable clinical outcomes of the TS-RECS group, including less blood loss (20,5–100 ml vs 95,10–310 ml, *P* < 0.0001) and better postoperative recovery in terms of the time to first oral intake (2,2–4 days vs 3,2–6 days, *P* < 0.0001) and postoperative hospital stay (5,4–10 days vs 8.5,5–16 days, *P* < 0.0001) compared with the LWR group. Moreover, preserving the intact gastric mucosa in TS-RECS could be attributed to another technical advantage over LWR. The importance of the intact mucosal layer during GSMT resection has been reported in some studies^20^,^21^. These studies considered that the intact mucosal layer could serve as a protective barrier between the gastric lumen (contaminated) and peritoneal cavity (clean), which could prevent peritoneal soiling during the procedure and reduce the risk of intra-abdominal infection and gastrointestinal tract leakage after surgery. In our matched cohort, although there was no significant difference in the overall incidence of postoperative complications between the two groups, there were two cases of leakage (Clavien–Dindo grade II and III) in the LWR group but none in the TS-RECS group, supporting the assertion that the integrity of the mucosal layer may prevent complications related to full-thickness resection.

In addition to these technical advantages, functional follow-up indicated that TS-RECS had the additional advantage of preserving gastrointestinal function. With advancements in surgical techniques and adjuvant therapy, the prognosis of patients with GSMTs, including GISTs, has been greatly improved, so gastrointestinal function after GSMT resection should be given more attention. Gastrointestinal function is closely associated with quality of life (QOL)^22^. Although the impact of local stomach resection on gastrointestinal function is far less significant than that of proximal or distal gastrectomy, we should not ignore this effect. Indeed, some patients who underwent GSMT resection experienced gastrointestinal symptoms after surgery, some of which even affected postoperative QOL. However, compared with the rich body of research focused on the technical feasibility of GSMT resection and survival prognosis, very few studies have assessed postoperative gastrointestinal function. To the best of our knowledge, the reported postoperative gastrointestinal function evaluations after resection of GSMTs, including endoscopic evidence of food residue, postoperative radioscopy, or postoperative body weight loss^9^,^23^,^24^, were not sufficiently comprehensive or specific. Recently, the GSRS questionnaire has been widely applied to evaluate gastrointestinal function and quality of life after gastrointestinal surgery^25^–^27^.

In the present study, we assessed postoperative gastrointestinal symptoms with a validated symptom-specific questionnaire, the GSRS, which could provide a comprehensive assessment to predict postoperative gastrointestinal function. In our functional follow-up results, the postoperative GSRS scores in the TS-RECS group, as well as the severity and frequency of gastrointestinal symptoms, were significantly decreased compared to those in the LWR group, especially for indigestion syndrome (borborygmus, eructation, abdominal distention and increased flatus). There are several possible explanations for the different postoperative gastrointestinal functions between the two groups. One might be vagal nerve injury. Given the important role of the vagal nerve in gastric motility and sensation regulation, vagal nerve injury may impair these functions and thereby lead to gastrointestinal symptoms^28^,^29^. Furthermore, several studies have reported that vagal nerve injury is associated with higher gastrointestinal symptom scores^28^,^30^,^31^. In the LWR group, stapler firings caused a considerable amount of wedge-shaped gastric wall to be resected along with the tumour, which could easily lead to unintentional injury to branches of the vagus nerve. However, the technical advantages of TS-RECS could facilitate less blood loss, more meticulous procedures and a smaller dissection area, which might decrease the risk of injury to important vessels and nerves (e.g., the vagus nerve). The other is the decreased gastric volume and gastric capacity. Some evidence suggests that gastric volume is closely correlated with gastrointestinal symptoms^32^. TS-RECS could resect GSMT with a minimal surgical margin and maximal preservation of the volume and capacity of the stomach, which could lead to greater preservation of postoperative gastrointestinal function.

However, there are some concerns regarding the TS-RECS technique. The oncological safety of a minimal margin is a major concern for two reasons. One reason is the technical difficulty of a minimal surgical margin. Removing the tumour with an intact pseudocapsule during the resection of GSMTs is of utmost importance. It is technically demanding to excise the tumour with a minimal R0 margin and simultaneously avoid rupture of the tumour pseudocapsule. The technical advantages of robotic surgery would facilitate these meticulous and demanding procedures and achieve a win–win outcome. In the present study, en bloc resection with minimal R0 margins was completed in all patients, and the intact tumour pseudocapsule was confirmed microscopically via postoperative pathology examination. The other reason is the oncological outcomes of patients with minimal surgical margins. Although a minimal surgical margin is not routinely recommended in surgical resection of GSMTs, an increasing number of studies with long-term follow-up have demonstrated that a minimal surgical margin for GSMT resection does not affect oncological outcomes^9^,^33^. In addition, the prognosis of patients with GSMTs was reported to be more dependent on tumour biology than on margin status^34^,^35^. Therefore, we believe TS-RECS could be used to achieve tumour resection with both complete oncological curability and maximal functional preservation. However, although our results showed some technical and function-preserving advantages in TS-RECS compared with LWR for GSMT resection, we should consider all aspects comprehensively when selecting a procedure, including patient characteristics, tumour properties and some socioeconomic factors (e.g., patient income), and try our best to help patients obtain the greatest benefit from the treatment.

There were some limitations to the current study that should be noted. First, although we used PSM to reduce the known selection bias and confounders, the single-centre retrospective design and small sample size led to some inherent limitations (e.g., insufficient statistical power, unknown selection bias and confounders). Second, because robotic surgery is more expensive than conventional laparoscopic surgery, although we have elaborated the potential advantages of TS-RECS, the cost-effectiveness of this technique needs to be further investigated. Finally, the median follow-up period was 18 months, which was not long enough to prove oncological validity. Undoubtedly, larger-scale prospective randomized controlled studies with long-term outcomes are needed in the future to further validate our findings. Nevertheless, to the best of our knowledge, this first is the study to provide a comprehensive assessment of the impact of surgical management of GSMT resection on postoperative gastrointestinal function.

In conclusion, our results indicated that TS-RECS for resection of GSMTs was associated with better surgical outcomes and more favourable functional outcomes than LWR. TS-RECS should be considered as a tailored minimally invasive surgery for resection of GSMTs that can preserve organ function.
